# Torsional stability of interference screws derived from bovine bone - a biomechanical study

**DOI:** 10.1186/1471-2474-11-82

**Published:** 2010-05-01

**Authors:** Joscha Bauer, Turgay Efe, Silke Herdrich, Leo Gotzen, Bilal Farouk El-Zayat, Jan Schmitt, Nina Timmesfeld, Markus Dietmar Schofer

**Affiliations:** 1 Department of Orthopaedics and Rheumatology, University Hospital Marburg, Baldingerstrasse, 35043 Marburg, Germany; 2 Department of Orthopaedics, Gotenstrasse 1-6, 65929 Frankfurt/Main, Germany; 3 Department of Trauma, Hand and Reconstructive Surgery, University Hospital Marburg, Baldingerstrasse, 35043 Marburg, Germany; 4 Institute of Medical Biometry and Epidemiology, Philipps-University Marburg, Bunsenstrasse 3, 35037 Marburg, Germany

## Abstract

**Background:**

In the present biomechanical study, the torsional stability of different interference screws, made of bovine bone, was tested. Interference screws derived from bovine bone are a possible biological alternative to conventional metallic or bioabsorbable polymer interference screws.

**Methods:**

In the first part of the study we compared the torsional stability of self-made 8 mm Interference screws (BC) and a commercial 8 mm interference screw (Tutofix^®^). Furthermore, we compared the torsional strength of BC screws with different diameters. For screwing in, a hexagon head and an octagon head were tested. Maximum breaking torques in polymethyl methacrylate resin were recorded by means of an electronic torque screw driver. In the second part of the study the tibial part of a bone-patellar tendon-bone graft was fixed in porcine test specimens using an 8 mm BC screw and the maximum insertion torques were recorded. Each interference screw type was tested 5 times.

**Results:**

There was no statistically significant difference between the different 8 mm interference screws (p = 0.121). Pairwise comparisons did not reveal statistically significant differences, either. It was demonstrated for the BC screws, that a larger screw diameter significantly leads to higher torsional stability (p = 9.779 × 10^-5^). Pairwise comparisons showed a significantly lower torsional stability for the 7 mm BC screw than for the 8 mm BC screw (p = 0.0079) and the 9 mm BC screw (p = 0.0079). Statistically significant differences between the 8 mm and the 9 mm BC screw could not be found (p = 0.15). During screwing into the tibial graft channel **of the porcine specimens**, **insertion **torques between 0.5 Nm and 3.2 Nm were recorded. In one case the hexagon head of **a **BC screw broke off during the last turn.

**Conclusions:**

The BC screws show comparable torsional stability to Tutofix^® ^interference screws. As expected the torsional strength of the screws increases significantly with the diameter. The safety and in vivo performance of products derived from xenogeneic bone should be the focus of further investigations.

## Background

The continuous increase in recreational sports leads to a continuously increasing number of capsule and ligament injuries of the knee joint as well. About 20% of the knee injuries are accompanied by anterior cruciate ligament (ACL) ruptures [1]. Every year, about 35,000 ACL ruptures occur in Germany, of which about 28,000 (80%) are treated surgically [2]. The reconstruction of the ACL belongs to the therapies of choice for the active patient and is one of most common ligament reconstructions in the knee [3]. Together with the semitendinosus and gracilis tendon, the central part of the patellar tendon with adjacent bone blocks has been proven to be a suitable transplant with high failure load and sufficient osteointegration [4,5]. The secure fixation of the transplant is required for the restoration of knee stability and early functional rehabilitation [6]. Thereby the tibial transplant fixation is of particular importance. The tibial fixation devices must resist higher shear forces applied parallel to the axis of the tibial drill channel [7,8]that has a lower bone density compared to the femur [9,10]. Laxdal et al. [11] showed that pull out of the tibial graft fixation was the most common reason for transplant failure in the early phase of rehabilitation.

Different methods of bone-patellar tendon-bone (BPTB) graft fixation are commercially available. The most common method for BPTB graft fixation is interference screw fixation [12]. Interference screws made of metal or absorbable polymers are available [4,13] and have specific advantages and disadvantages. Metal screws are implanted permanently and can lead to complications at the implant site or have to be removed sometimes in a second operation - which can be difficult and time consuming [14]. Additionally, metal implants cause artefacts in MRI examinations and thus may complicate further diagnostics. In the case of bioabsorbable polymer screws the degradation process is still a problem [15] and the course of degradation is highly variable [16,17]. Screws made of bioabsorbable materials can break at high torque during implantation [18]. Broken screws are difficult to adjust or to remove, if they are not positioned ideally when breakage occurs. Screw fragments can impair the graft and cause intraarticular damage [19,20].

Interference screws derived from bovine compact bone are a biological alternative to conventional materials [21]. The clinical application of bovine cancellous bone as bone graft or implant in orodental surgery has already been reported [22,23]. Ideally, xenogeneic materials are firmly incorporated into the bone and are substituted over the course of time by autologous bone [23]. In previous studies, the maximum failure load [24-31] and the torsional stability [18,30,32-34] of interference screws have been investigated. In these studies, screws derived from xenogeneic material did not get much attention. To our knowledge, there is no biomechanical study so far regarding the torsional strength of xenogeneic interference screws.

In the first part of the study we evaluated the biomechanical properties of self-made interference screws (BC) as well as an industrial produced interference screw (Tutofix^®^) in polymethyl methacrylate resin. The aim of this part of the study was to provide data for maximum breaking torques of the 8 mm BC and 8 mm Tutofix^® ^interference screws and compare them with the results of bioabsorbable polymer screws provided in the literature. Furthermore, we provided data for maximum breaking torques of BC screws with different diameters. We hypothesised that the maximum breaking torques of the different 8 mm interference screws differ significantly and that a larger diameter significantly correlates with a higher maximum breaking torque. In the second part of the study we investigated the biomechanical properties of the 8 mm BC screw in a porcine tibia. The aim of this part of the study was to provide data for the insertion torque and compare with the results of bioabsorbable polymer screws provided in the literature. There is little data on bovine interference screws. Testing their mechanical properties with two different methods seems to be appropriate to evaluate their value in a possible clinical setup.

## Methods

Interference screws derived from bovine bone were tested. At the beginning of the investigation, commercial screws of bovine bone were only available with a diameter of 8 mm. The limited availability forced us to produce screws with diameters of 7 mm, 8 mm and 9 mm. Five specimens of each type of interference screw were tested. All experiments were carried out by the same person to minimise differences in the test performance.

### Interference screws

**Figure 1:**
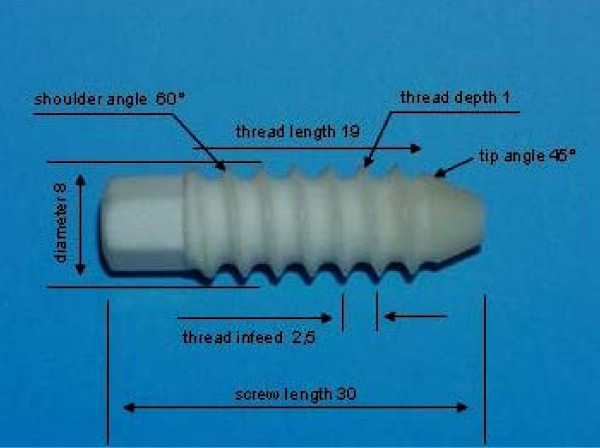
**Design specification of the 8 mm BC interference screw with hexagon head**.

The BC interference screws were produced from cortical bone of bovine tibial diaphyses by the department of precision mechanics of our institution (Fig. 1). Longish segments of 7 × 30 mm, 8 × 30 mm and 9 × 30 mm were cut out of the cleaned bone shafts and cylindrically turned on a lathe (Hommels-Herkules, EBK 450, Viernheim, Germany). Subsequently the screw thread was cut (thread depth 1 mm, thread infeed 2.5 mm per turn). The non-cannulated screws had a hexagonal drive head. The elongated thread free tip was intended to ensure a target-oriented implantation without a guide wire. The 8 mm BC screw was also tested with an octagonal drive head. In pre-test other insertion devices had shown to be unstable and not feasible. As the screws did not have a full thread, the thread length was 19 mm. After completion, the screws were treated in a 99% acetone bath to extract lipids, to kill microorganisms and to reduce the antigenic properties. The screws were autoclaved at 121°C and 3 bar steam pressure for 20 min and stored at room temperature in a dry place until they were used. The 8 × 21 mm Tutofix^® ^screw (Tutogen, Neunkirchen am Brand, Germany) had a thread length of 18 mm, a thread depth of 0.7 mm, a thread infeed of 2.1 mm per turn and a hexagonal drive head. The screws were processed according to the Tutoplast^® ^process. The Tutoplast^® ^process is a validated bone sterilisation method and is certified for bovine bone. Biocompatibility and biomechanical integrity have been demonstrated [35-37].

### Torsional stability in polymethyl methacrylate

**Figure 2:**
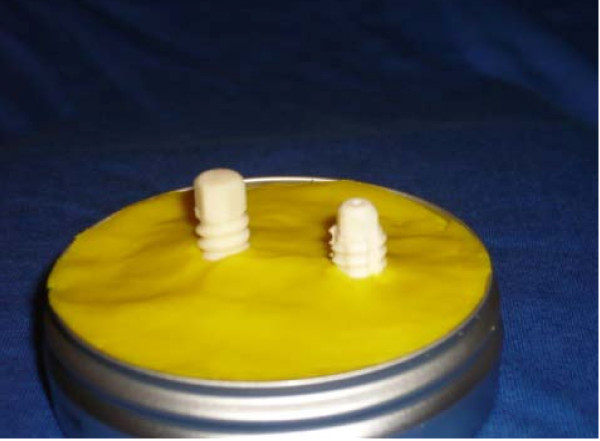
**Interference screws derived from bovine bone in polymethyl methacrylate, left BC screw, right Tutofix^® ^screw**.

**Figure 3:**
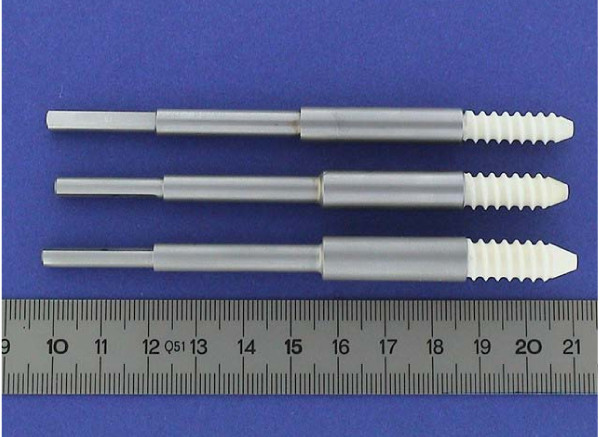
**7 mm, 8 mm and 9 mm BC interference screws with screw driver adapter**.

24 hours before testing, the screws were embedded into a 20 mm layer of polymethyl methacrylate resin (Heraeus, Technovit 9100, Wehrheim, Germany). The screws protruded 10 mm from the resin surface (Fig. 2) to simulate a worst-case situation in vivo, in which the screws jam during screwing in at the graft channel. For each type of screw, a custom-made adapter (Fig. 3) was used, which was mounted onto the electronic torque screw driver (Totti, DTDK-N50 E, Wuppertal, Germany). The screw driver was calibrated from 0.5 Nm to 5 Nm and had a measuring accuracy of 0.005 Nm. On the day of the experiment, the polymethyl methacrylate resin with the embedded screws was firmly clamped into a holder. The screws were slowly turned manually under a constant axial loading until screw breakage occurred. Maximum breaking torque was recorded for each screw.

### Torsional stability with porcine knee specimen

Porcine specimen (age 12-14 month) were supplied by the local slaughterhouse on the day of slaughter and then frozen at -20°C. Prior to the experiments the test specimen were thawed slowly for 13 hours at room temperature. A 10 mm BPTB graft was obtained according to standard procedure and the tibial bone plug was cut to 25 × 10 × 7 mm. Using a cruciate ligament targeting device (Synthes, Umkirch, Germany), a Kirschner wire was applied and the graft channel was reamed to 10 mm. The angle between the drill channels and the tibial long axis was 50°. A thread was cut into the drill channel and the grafts fixed with an 8 mm BC screw (hexagon head) under a pretension of 60 Newton (N). The screws were placed directly below the articular surface always at the same relative position between the graft and the tunnel. Insertion torques were recorded with an electronic torque screw driver (Totti, DTDK-N50 E, Wuppertal, Germany).

### Statistical analysis

Descriptive analysis was performed by determination of mean values, standard deviations, minimum and maximum values. To investigate the difference between the three 8 mm screws, a non-parametric analysis of variance (Kruskal-Wallis test) was performed. To analyse the association between diameter and breaking torque, the Jonckheere-Terpstra test for trend was performed. All pairwise comparisons were done by the Wilcoxon rank-sum test. The significance level was set to P < 0.05.

## Results

**Figure 4:**
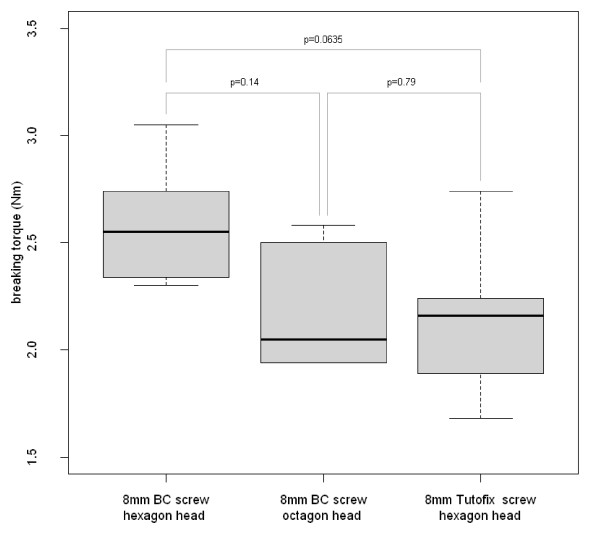
**Pairwise comparison of the maximum breaking torques of three different 8 mm interference screws in polymethyl methacrylate resin and p-values (Kruskal-Wallis test)**. There are no statistically significant differences between the three tested screw types

**Figure 5:**
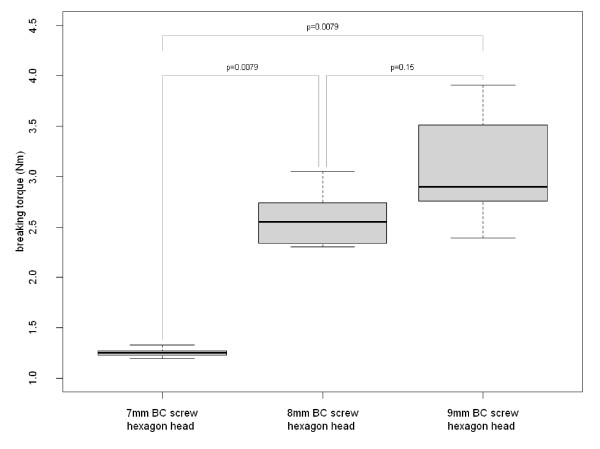
**Pairwise comparison of the effect of the screw diameter on maximum breaking torques of BC interference screws in polymethyl methacrylate resin and p-values (Jonckheere-Terpstra trend test)**. There is a statistically significant difference between 7 mm screws and the two larger screw types. Statistically significant differences between 8 mm and 9 mm BC screw could not be found

Regarding the torsional stability, there was no statistically significant difference between the three types of 8 mm interference screws (p = 0.121). Fig. 4 shows the pairwise comparison of the three different 8 mm interference screws. A larger screw diameter significantly corresponded with an increasing torsional stability (p = 9.779 × 10^-5^). Fig. 5 shows the pairwise comparison of three different screw diameters. At the graft channel, the mean insertion torque was 0.58 ± 0.08 Nm at the beginning of screwing in. At the last turn until complete screw implantation, the mean insertion torque was 3.0 ± 0.33 Nm. During the final rotation the maximum value was 3.31 Nm, the minimum value was 2.50 Nm. One hexagonal head broke off during the last turn of screwing in at 3.20 Nm.

## Discussion

The results of the present study partly confirmed the hypotheses postulated in the beginning. It could not be confirmed that the torsional stabilities of the three different 8 mm interference screws differ significantly. It was demonstrated for the different BC screws that a larger screw diameter leads to higher torsional stability. Pairwise comparisons confirmed the hypothesis that the torsional stability of the 7 mm BC screw was lower than that of the 8 mm BC screw and the 9 mm BC screw. The postulated hypothesis regarding the pairwise comparison between 8 mm and 9 mm BC screw could not be confirmed.

The biomechanical properties of interference screws made of metal and bioabsorbable polymer materials have been featured in numerous experimental studies which focussed mainly on pull-out testing [26,38-42]. Only a limited number of studies dealt with torsional stability of interference screw. Costi et al. [32] tested the torsional stability of 5 different bioabsorbable interference screws. Half the length of the 20 mm screws was embedded in polyurethane resin. The screws were turned manually with an electronic screw driver until failure occurred. The mean failure torque was between 1.07 ± 0.18 Nm (7 mm poly-(glycolide-co-trimethylene carbonate)) and 5.23 ± 0.24 Nm (8 mm poly-(D, L-lactide)). Furthermore, they noticed that the screw diameter had a significant effect on failure torque.

Weiler et al. [30] determined maximal torque at failure for bioabsorbable interference screws between 2.30 ± 0.10 Nm (8 mm poly-(glycolide-co-trimethylene-carbonate)) and 9.06 ± 0.68 Nm (7 mm poly-(D, L-lactide)). They also found that the torsional stability is highly depending on the drive design. In a cadaver study Kohn and Rose [43], investigated the influence of screw diameter and insertion torque on primary stability. They found that tibial fixation using 9 mm screws was significantly stronger than tibial fixation using 7 mm screws. Comparing the results of the present study to the values Costi et al. [32] acquired, we demonstrated that the BC screws have a torsional stability equal to that of most bioabsorbable screws. Especially the 8 mm BC screw with a hexagonal head and the 9 mm screw were stronger than most bioabsorbable screws. Comparing the results of the present study to those reported by Weiler et al. [30] the stability of the BC screws appeared to be lower than that of the bioabsorbable interference screws tested. A possible explanation for the lower breaking torques of the BC screws in the present study is that in the experimental setup of Weiler et al. [30] the screws protruded only 1 mm from the polyurethane. Additionally the use of polymethyl methacrylate resin in the present study might be a reason for the lower stability. During the hardening of polymethyl methacrylate, higher temperatures arise than with polyurethane resin [30]. This can cause a change of shape as well as destabilisation of the BC screws.

Bovine compact bone interference screws as well as bioabsorbable interference screws [44] can break off during screwing in. This usually happens in the final phase of screw insertion, when the highest torque values are reached. Piltz et al. [45] determined a mean insertion torque of 1.6 ± 1.1 Nm for BPTB graft fixation at the tibial channel with bioabsorbable interference screws (8 mm poly-L-lactide). In the results of other studies, values from 0.30 ± 0.19 to 0.60 ± 0.30 Nm (7 mm poly-(L-lactide)) [18] and from 0.71 ± 0.14 Nm (9 mm poly-(D, L-lactide co-glycolide)) to 2.45 ± 0.66 Nm (8 mm poly-(L-lactide)) [30] are reported. In the present study a mean insertion torque of 1.83 ± 0.76 Nm was applied for the 8 mm BC screw. The self-made BC screws showed a good torsional stability and were able to withstand the usual intraoperative loads. Biomechanical studies can provide insight into the torsional stability of interference screws with different diameters and designs. However, conclusions about clinical applications are difficult to draw, as in vivo additional bending and shear forces act upon the screws. Furthermore, the results of biomechanical studies depend on a variety of factors such as bone quality, tissue species (human, xenogeneic) and screw design, size and material [30]. Therefore, a direct comparison of the study models in the literature is possible only to a certain extent. However, the constant experimental conditions allow a comparison of the fixation elements' performance in each test model.

We observed one BC screw breakage at the end of the insertion in the graft tunnel in porcine test specimens. In most cases bioabsorbable screws have screwdriver holes that run the entire length of the screw, thus when breakage occurs in many cases the whole screw is damaged by splitting [32]. The BC screws have an external head for the screw driver that functions as a predetermined breaking point. The screw head breaks off under maximal load usually at the end of the insertion. In order to reduce the number of screw breakages, the load transfer between screw driver and screw should be improved in future design modifications. Due to the fact that BC screws are not cannulated, the risk of malpositioning is increased. Although it is technically possible to produce cannulated bone screws, early tests showed them to be too fragile. The BC screws had a longer non-threaded tip in order to minimise diversity and provide a standardised, reproducible placement. In other biomechanical studies non-cannulated screws were examined and were implanted in the same way [30].

Despite the good clinical results of the BPTB [46] and soft tissue interference screw fixation [47] complications have been reported. Screw thread laceration of the bone plug, the tendon itself or the graft traction sutures are clinically important and may be reasons for failure [48,49]. Particularly, there are concerns about the soft tissue graft damage by the screw threads. Zantop et al. [50] could show by direct comparison of bioabsorbable and titanium interference screws, that graft damage was significantly higher in the titanium interference group. This is due to the fact that titanium screws had sharp thread edges. In theory, it is possible to minimise graft damage by using interference screws with rounded thread edges. The thread edges of both the BC and the Tutofix^® ^are rounded, but it is not possible to conclude from the present study, if rounded thread edges still can cause transplant damage.

The ideal interference screw material should be replaced by cancellous autologous bone. In a prospective study by Tecklenburg et al. [51], BPTB grafts were used in 40 patients for ACL reconstruction. Tibial fixation was carried out with two conventional bioabsorbable interference screws (poly-(L-lactide/hydroxyapatite)) and poly-(L-lactide/β-tricalcium phosphate)) and with an allogeneic interference screw. After a follow-up of 24 months there were no significant differences between the different screws regarding the subjective and the clinical results. MRI showed that only the allogeneic screws were incorporated completely. Complete incorporation of xenogeneic materials was proven by different authors [23,52]. The microstructure of xenogeneic bone resembles that of human bone [53]. There is no difference between bovine and human material regarding osteoblast proliferation on dehydrated cancellous bone slices [54]. However, some possible problems concerning xenogeneic implants like immune reaction must be taken into account and have to be investigated in further studies. In theory, cellular components are removed from the implants by validated sterilisation methods [55]. The bovine spongiform encephalopathy (BSE) has shown that it is possible that previously unknown pathogenic agents like prions are not inactivated completely by conventional methods.

A possible methodical weakness of the present study may be the manual screw turning. If the experiment is performed inappropriately, high bending moments arise by the screw driver. To minimise disturbing factors like this, all experiments were carried out by the same person, and attention was paid that a constant axial loading without bending was applied. The manual turning, however, corresponds to the in vivo situation during surgery, where the mentioned bending moments can occur in the same way. A further drawback of our study is the low number of screws tested. In comparative studies, the number of screws tested is equally [30,56] or slightly higher [32]. The small number of tested screws does not allow a general conclusion about the biomechanical properties of interference screws derived from bovine compact bone. Bigger numbers might have been preferable. Nevertheless, the results presented in this study might serve for later projects with this model.

The availability of young human knees for test models is often limited. Therefore, it is reasonable under experimental aspects to use porcine test specimens, because porcine knee joints resemble the human knee joint anatomically [57] and are a well acknowledged animal model [13]. It could be shown that the average bone density in the proximal porcine tibia was similar as in the proximal tibia of young human bone [58]. Brown et al. [59] noticed that animal specimens are more appropriate for studies than specimens from older humans.

The search for an ideal interference screw material for ACL graft fixation is still on-going. The material should be incorporated completely and be replaced fully by endogenous cancellous bone. This would also be helpful in revision cases where sometimes due to bone resorption two operations are necessary.

## Conclusion

The results of the present study showed no significant differences regarding the torsional stability between the three different 8 mm interference screws. A larger screw diameter significantly lead to higher torsion stability. Further improvement of the design of the screws and the instruments should also improve the biomechanical properties of BC screws. The safety and in vivo performance of products derived from xenogeneic bone should be the focus of further investigations.

## Competing interests

The authors declare that they have no competing interests.

## Authors' contributions

Each author has made substantial intellectual contributions to this study: JB: performed the experiments, drafted the manuscript; TE: performed the experiments, drafted and revised the manuscript; SH and NT: performed the statistical analysis; LG: initiated the study, and participated in its design and coordination; BFE, JS, MDS: participated in analysis and interpretation of data. All authors read and approved the final manuscript.

## Pre-publication history

The pre-publication history for this paper can be accessed here:

http://www.biomedcentral.com/1471-2474/11/82/prepub
